# Salmonid alphavirus infection causes skin dysbiosis in Atlantic salmon (*Salmo salar* L.) post-smolts

**DOI:** 10.1371/journal.pone.0172856

**Published:** 2017-03-06

**Authors:** Kristin M. Reid, Sonal Patel, Aaron J. Robinson, Lijing Bu, Jiraporn Jarungsriapisit, Lindsey J. Moore, Irene Salinas

**Affiliations:** 1 Department of Biology, University of New Mexico, Albuquerque, New Mexico, United States of America; 2 Institute of Marine Research, Bergen, Norway; INRA, FRANCE

## Abstract

Interactions among host, microbiota and viral pathogens are complex and poorly understood. The goal of the present study is to assess the changes in the skin microbial community of Atlantic salmon (*Salmo salar L*.) in response to experimental infection with salmonid alphavirus (SAV). The salmon skin microbial community was determined using 16S rDNA pyrosequencing in five different experimental groups: control, 7 days after infection with low-dose SAV, 14 days after infection with low-dose SAV, 7 days after infection with high-dose SAV, and 14 days after infection with high-dose SAV. Both infection treatment and time after infection were strong predictors of the skin microbial community composition. Skin samples from SAV3 infected fish showed an unbalanced microbiota characterized by a decreased abundance of Proteobacteria such as *Oleispira sp*. and increased abundances of opportunistic taxa including *Flavobacteriaceae*, *Streptococcaceae* and *Tenacibaculum sp*. These results demonstrate that viral infections can result in skin dysbiosis likely rendering the host more susceptible to secondary bacterial infections.

## Introduction

The mucosal surfaces of animals are at the interface between the host and the environment. At these surfaces, millions of microorganisms form an intimate and successful relationship with the host. Both environmental factors and host characteristics shape the composition of microbiomes.

Teleost fish have diverse microbial communities associated with each mucosal body site. Environmental factors known to influence teleost microbiomes include: diet, stress, water quality, toxicants, and infection [1, 2, 3, 4, 5].

During the course of an infection, the relationships between the microbiota and the host immune system are vulnerable to changes. Additionally, the microbiota-host immune system interaction largely impacts the outcome of an infection [6]. Thus, three-way interactions between pathogens, microbiota, and the animal host are complex and require a deep understanding of all three components. A number of studies have reported changes in the microbiome of farmed teleost fish as a result of parasitic [7] or bacterial infection [8, 9], but how viral infections affect teleost microbiota is unknown.

The microbiota is known to protect mammalian hosts from viral infections [10]. However, in some cases, microbiota can also facilitate viral infection and propagation [11, 12, 13, 14]. Very little is known about the effects of viral infections on the microbiota of any animal host. HIV-infected humans show a state of dysbiosis in their gut microbiota [15] and Influenza-virus infection alters the intestinal microbial community of mice [16].

Atlantic salmon (*Salmo salar* L.) is one of the most important commercial aquaculture fish species. Atlantic salmon are susceptible to a number of disease agents including parasites, bacteria and viruses. Currently, one of the major threats to salmonid culture in Europe is pancreas disease caused by salmon alphavirus (SAV). There are six different SAV subtypes (SAV1-6) that are all members of the genus Alphavirus within the family *Togaviridae* [17, 18], and the subtype SAV3 has only been shown to cause pancreas disease outbreaks in Norway in seawater phase of the salmon life-cycle. SAV targets several internal organs including the pancreas, heart and muscle. Impact of infection on haematopoetic tissue has also been reported [18]. Importantly, infected salmon shed viral particles via their feces and skin mucus [19, 20]. Thus, the goal of this study was to investigate the changes in the bacterial microbial community present in the skin of Atlantic salmon experimentally infected with SAV3 by bath immersion.

## Materials and methods

### Animals

Samples used in this study were derived from a recently published study [21]. The experiment was carried out at the Industrial and Aquatic Laboratory (ILAB), Bergen High Technology Centre, Bergen, Norway using unvaccinated post-smolts raised by ILAB. The fish strain was from SalmoBreed, Osterøy, Norway. The experimental fish with an average weight of 50.6 ± 6.8 g and an average length of 16.3 ± 0.8 cm were kept in seawater (34‰) at 12°C. The seawater flow rate and oxygen saturation were maintained at 300L h^−r^ and >80%, respectively. All handling procedures were performed under metomidate (10 mg L^−1^) and benzocaine (60 mg L^−1^) anaesthesia, or metomidate (10 mg L^−1^) and benzocaine (160 mg L^−1^) were used for euthanasia.

### Virus, experimental infection, and sampling

The bath challenge model with SAV3 in seawater was carried out as described previously [22] with some modifications. Briefly, one hundred and eighty fish were injected intra-muscularly with 100 μl SAV3 containing 10^3^ TCID_50_ and divided equally into three rectangular 150-L tanks for production of virus into the seawater. On the day of the bath challenge, the water flow to the three shedder tanks was stopped for 1 hour with a supply of aeration, after which the shedder fish were removed and euthanized. The seawater containing SAV3 (SAV3-seawater) from all three shedder tanks was mixed to prepare each of the dilutions of SAV3-seawater and the fish were bathed for 6 h in 120 L of the respective SAV3 doses. Fish exposed to either a 1:75 dilution of SAV3-seawater (low-dose) or undiluted SAV3-seawater (high-dose) corresponding to 7 and 139 TCID_50_ SAV3 L^-1^ of seawater, respectively were used in the present study [21]. To check the prevalence of SAV3 in the exposed individuals, half the heart of each individual was collected and snap frozen in liquid nitrogen as explained below. In addition, viral shedding into the seawater from the exposed groups was measured confirming the presence of infection and viraemic period in these groups [21]. A triangle of skin approximately 2 cm^2^ was collected from above the lateral line and below the dorsal fin into 1 ml of selective lysis buffer (SLB buffer) [23]. Skin samples from eight fish exposed to low and high doses were sampled at 7 and 14 days post-exposure and stored immediately on dry ice. This study was approved by the Norwegian Animal Research Authority (NARA) and carried out in strict accordance with the guidelines.

### RNA extraction, cDNA synthesis and RT-qPCR analysis for SAV nsP1

Total RNA was extracted from heart tissue with TRIzol^®^ reagent (Ambion) and an iPrep^™^ PureLink^™^ Total RNA Kit (Invitrogen, USA) according to the manufacturer’s instructions and quantified using a NanoDrop ND-1000 spectrophotometer (Thermo Scientific). Two hundred ng of total RNA isolated from heart samples was used in cDNA synthesis in a 10 μL reaction volume (SuperScript VILO cDNA Synthesis Kit). A 1:10 dilution of cDNA was then used in a qPCR assay targeting the SAV nsP1 gene for detection of SAV3 [24]. The 2 μL of diluted cDNA was used in qPCR reaction mixture (TaqMan^®^ Fast Universal Master Mix (Applied Biosystems^®^) with 900 nM each of forward and reverse primers, and 250 nM of probe in a total volume of 10 μL on 384 well-plates. The qPCR assay was performed using ABI 7900HT Fast Real-Time PCR system (Applied Biosystems) and the temperature profile was adjusted as follows; activation at 95°C for 20 s followed by 40 cycles of denaturation at 95°C for 10 s, and annealing and extension at 60°C for 20 s. A threshold value of 0.1 was applied to all samples.

### DNA isolation, bacterial 16S rRNA gene PCR amplification, and pyrosequencing

Total genomic DNA was extracted from skin samples, including both fish and bacterial DNA. Sterile 3-mm tungsten carbide beads (Qiagen) were used to homogenize the tissue samples in a TissueLyser II (Qiagen). For extraction, we followed the cetyltrimethylammonium bromide (CTAB) buffer method as previously described [23]. DNA pellets were then resuspended in 30 μl of DNase- and RNase-free molecular biology grade water. Sample DNA concentration and purity was measured in in a NanoDrop ND 1000 (Thermo Scientific).

Bacterial community composition in skin samples was determined by pyrosequencing of prokaryotic16S rRNA genes. Total genomic DNA for each sample was diluted 1 in 10 or 1 in 100 in RNAse free water and amplified in triplicate using Illumina adapter fused primers that target V1-V3 variable regions of the prokaryotic 16S rRNA sequences. Gene specific primer sequences used were: 28F 5’-GAGTTTGATCNTGGCTCAG-3’ and 519R, 5’-GTNTTACNGCGGCKGCTG-3’ (where N = any nucleotide, and K = T or G). All DNA samples were diluted 1:100 or 1:10 for PCR amplification. The amplification was carried out with initial activation of the enzyme at 94°C for 90s followed by 33 cycles of the following: 94°C for 30s, annealing at 52°C for 30s, and 72°C for 90s, and a 7 min extension cycle at 72°C with a final holding temperature of 4°C. PCR amplicons were purified using Axygen AxyPrep Mag PCR Clean-up Kit (Fisher Scientific) as per manufacturer’s instructions. Samples were then indexed by ligating index barcode to Illumina adapters onto the PCR amplicon using the Nextera XT Index Kit v2 Set A (Illumina). DNA concentrations in each sample were quantified, pooled and adjusted to a DNA concentration of 200ng/μl. Pooled samples were purified again using the Axygen AxyPrep Mag PCR Clean-up Kit and sequenced in Illumina MiSeq platform using the MiSeq^®^ Reagent Kit v3 (600 cycle) at the Clinical Translational Science Center at University of New Mexico Health Sciences Center.

### Sequence analysis

Sequence data was analysed using Quantitative Insights Into Microbial Ecology (QIIME 1.9) pipeline [25] within the web-based platform Galaxy at the University of New Mexico [26]. Operational Taxonomic Units (OTUs) were selected by open reference picking using sumaclust method. OTUs were aligned to the version 123 (last updated May, 20 2016) of the SILVA 16S/18S database with a 97% identity level. In order to generate rarefaction curves and assess sampling depth, rarefaction analysis was performed in QIIME using several alpha diversity metrics (PD_whole_tree, chao1, and observed_otus). Four samples were discarded due to low sampling depth. Core diversity analysis was run on the remaining samples with a normalized sampling depth of 5,800 sequences. Alpha diversity metrics included Shannon diversity index, chao1, PD, Good’s coverage, and number of OTUs. Non-phylogenetic and phylogenetic beta-diversity analyses were performed in QIIME using the Bray Curtis metric or the unweighted and weighted UniFrac, respectively. Principal coordinate analysis and taxonomic summaries were produced in QIIME to compare the bacterial community in all five experimental groups.

### Statistical analysis

Two-way analysis of variance (ANOSIM) was performed in R using treatment and time after infection as the variables. Differences were considered statistically significant when P<0.05.

## Results

### Sequencing results

All samples were run in a single Illumina MiSeq run. We obtained a total of 18314985 sequencing reads for the original 40 samples. After removal of samples with low read numbers, the 36 samples use for downstream analysis had a total of 9688827 reads with a mean read number per sample of 269134 ± 268730. Pair end merging and quality trimming resulted in a total of 7192382 post-filter sequencing reads. The mean number of post-filter reads per sample was 199788 ± 197425 and the amplicon size was 301 bp.

### SAV infection results in changes in the alpha-diversity of salmon skin microbial community

**Fig 1:**
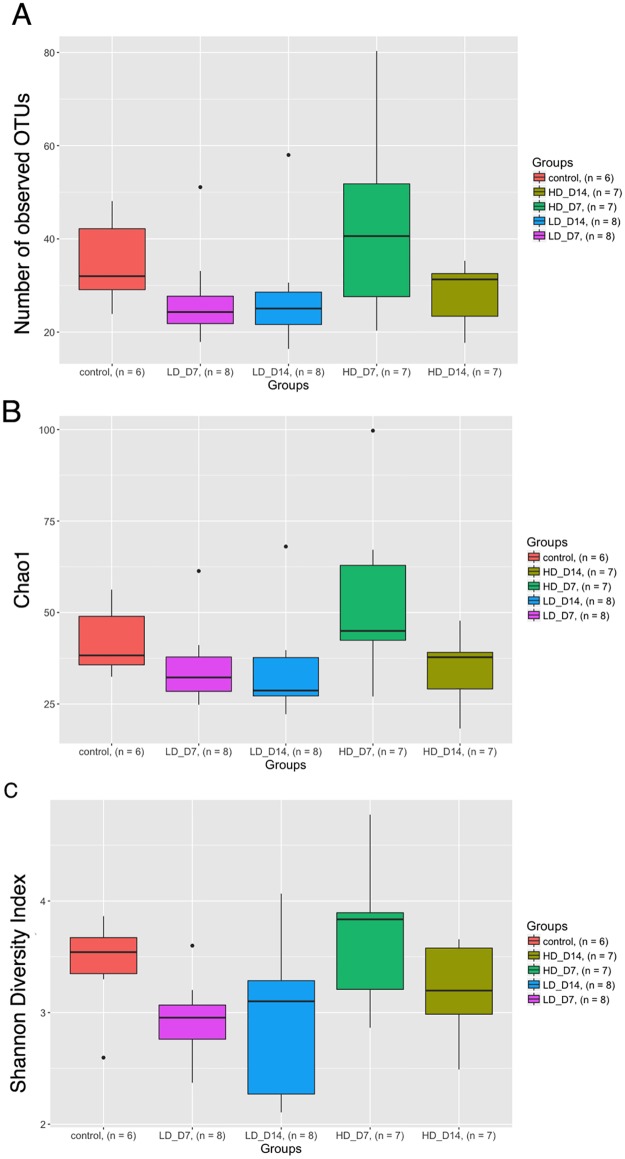
Changes in the alpha diversity of the Atlantic salmon skin microbial community in response to SAV3 infection. A) Total number of OTUs. B) Chao1. C) Shannon diversity index. LD_D7: low-dose infected day 7; LD_D14: low-dose infected day 14; HD_D7: high-dose infected day 7; HD_D14: high-dose infected day 14.

The total number of different OTUs detected in the low-dose infected experimental groups day 7 and day 14 was lower than in control group skin samples (Fig 1A, Table 1). Similarly, the Chao1 diversity index of both low dose infected groups was significantly lower than the rest of the treatments (Fig 1B). For Shannon diversity index, the low dose day 7 group had a lower value than the rest of the treatments. Overall, the high-dose day 7 infected group had increased alpha diversity values (Fig 1A–1C) than the rest of the experimental groups. None of these differences were statistically significant.

**Table 1:** Alpha diversity metrics (mean ± standard deviation) of the salmon skin microbial community of the five experimental groups in this study.

Group	observedOTUs	Chao1	Shannon	PDwhole tree	Goods coverage
Control	34.66 ± 8.93	40.62 ± 9.42	3.42 ± 0.41	3.42 ± 0.71	0.999 ± 0.000212
LD_D7	27.61 ± 9.57	36.97 ± 9.62	2.95 ± 0.33	3.42 ± 0.38	0.998 ± 0.000662
LD_D14	28.26 ± 11.54	34.79 ± 13.75	2.93 ± 0.63	3.22 ± 0.69	0.999 ± 0.000309
HD_D7	42.82 ± 19.70	54.58 ± 25.1	3.66 ± 0.61	4.26 ± 1.41	0.998 ± 0.000662
HD_D14	28.17 ± 6.81	37.38 ± 13.21	3.21 ± 0.41	3.51 ± 1.15	0.999 ± 0.000425

### SAV infection results in skin microbial dysbiosis

In order to identify bacteria contributing to the changes in alpha diversity in the SAV-infected groups, we classified the phylum, order, family, and genus of the sequences in control and infected groups. A total of 18 OTUs showed significantly different abundances among treatments (Table 2).

**Table 2:** List of OTUs with significantly different abundances among experimental groups in this study.

OTU	Test-Statistic	P	FDR_P	Bonferroni_P	control	LD_D7	LD_D14	HD_D7	HD_D14	Taxonomy
New.ReferenceOTU13	20.084	0.0005	0.185	0.198	445.833	0.250	0.000	35.143	0.000	Proteobacteria; Gammaproteobacteria; Oceanospirillales; Oceanospirillaceae;
New.ReferenceOTU22	18.585	0.001	0.185	0.390	0.000	155.875	0.000	540.143	462.857	Bacteroidetes; Flavobacteriia; Flavobacteriales; Flavobacteriaceae; Ulvibacter;
4458669	17.806	0.001	0.185	0.554	3.500	0.000	0.000	0.143	0.000	Proteobacteria; Gammaproteobacteria; Oceanospirillales; Oceanospirillaceae;
835189	15.873	0.003	0.263	1.000	212.500	0.000	0.000	0.000	0.000	Proteobacteria; Gammaproteobacteria; Oceanospirillales; Oceanospirillaceae; Oleispira;
New.ReferenceOTU37	15.873	0.003	0.263	1.000	60.500	0.000	0.000	0.000	0.000	Proteobacteria; Gammaproteobacteria; Alteromonadales; Alteromonadaceae; BD2-13;
4396362	15.124	0.004	0.305	1.000	467.167	0.125	0.000	2.286	0.000	Proteobacteria; Gammaproteobacteria; Oceanospirillales; Oceanospirillaceae; Oleispira;
New.ReferenceOTU1	12.727	0.013	0.583	1.000	568.667	0.125	0.125	6.714	0.000	Proteobacteria; Gammaproteobacteria; Oceanospirillales; Oceanospirillaceae; Oleispira;
547302	11.554	0.021	0.583	1.000	144.833	0.000	0.000	6.429	0.000	Proteobacteria; Gammaproteobacteria; Oceanospirillales; Oceanospirillaceae; Marinomonas;
830290	11.114	0.025	0.583	1.000	41.833	0.000	0.000	38.143	0.000	Proteobacteria; Gammaproteobacteria; Vibrionales; Pseudoalteromonadaceae; Pseudoalteromonas;
806640	10.764	0.029	0.583	1.000	7.833	0.000	0.000	110.286	0.000	Proteobacteria; Gammaproteobacteria; Alteromonadales; Colwelliaceae;
New.ReferenceOTU38	10.294	0.036	0.583	1.000	1.667	0.000	0.000	0.000	0.000	Proteobacteria; Gammaproteobacteria; Oceanospirillales; Oceanospirillaceae; Oleispira;
197286	10.286	0.036	0.583	1.000	54.333	0.000	0.000	0.000	0.000	Proteobacteria; Gammaproteobacteria; Enterobacteriales; Enterobacteriaceae;
4376233	10.286	0.036	0.583	1.000	0.500	0.000	0.000	0.000	0.000	Proteobacteria; Gammaproteobacteria; Enterobacteriales; Enterobacteriaceae;
166927	10.286	0.036	0.583	1.000	1.833	0.000	0.000	0.000	0.000	Proteobacteria; Alphaproteobacteria; Rhodobacterales; Rhodobacteraceae;
New.ReferenceOTU48	10.286	0.036	0.583	1.000	43.667	0.000	0.000	0.000	0.000	Proteobacteria; Alphaproteobacteria; Rhodobacterales; Rhodobacteraceae;
509773	9.985	0.041	0.583	1.000	0.000	0.000	0.500	7.143	0.000	Firmicutes; Bacilli; Lactobacillales; Streptococcaceae; Streptococcus;
New.ReferenceOTU61	9.958	0.041	0.583	1.000	0.000	0.125	0.000	3.571	4.857	Bacteroidetes; Flavobacteriia; Flavobacteriales; Flavobacteriaceae; Tenacibaculum;
4306177	9.475	0.050	0.583	1.000	0.000	0.000	0.250	3.857	0.000	Firmicutes; Bacilli; Lactobacillales; Streptococcaceae; Streptococcus;

At the phylum level, the mean abundance of Proteobacteria dropped from 41.2% in controls to 11–22% in infected individuals. The lowest abundance of this phylum was found in the low-dose day 7 experimental group (Fig 2). Additionally, the abundance of Firmicutes present dropped from 15% in controls to 6–7% in all infected groups. Bacteroidetes abundance, in turn, increased from 3% in controls to 16–18% in the high-dose infected groups. In the low-dose infected groups, an increase in Bacteroidetes abundance was observed on day 7, but not on day 14. Fusobacteria abundance also increased as a result of infection from ~ 20% in controls to ~ 46% and ~ 30% in the low-dose and high-dose day 14 groups, respectively. Finally, Actinobacteria abundance also increased as a result of SAV infection with the greatest increases occurring on day 7 in both infected groups.

**Fig 2:**
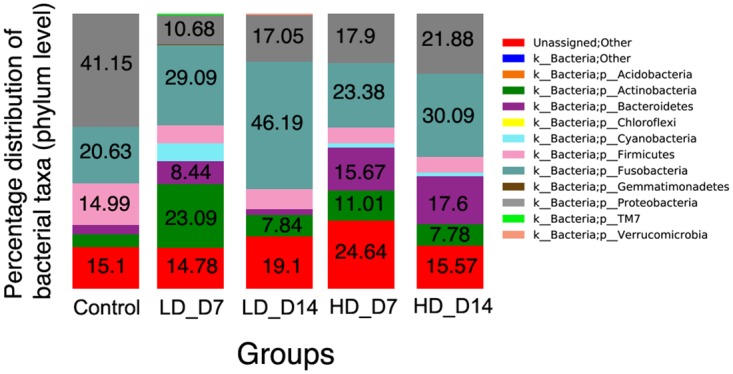
Bacterial community composition of Atlantic salmon skin in each experimental group. Bar chart of the mean relative abundance of phyla present in the salmon skin from the five different experimental groups. LD_D7: low-dose infected day 7; LD_D14: low-dose infected day 14; HD_D7: high-dose infected day 7; HD_D14: high-dose infected day 14. Number represent the relative percentage of each phyla that had abundances greater than 7.6%.

**Fig 3:**
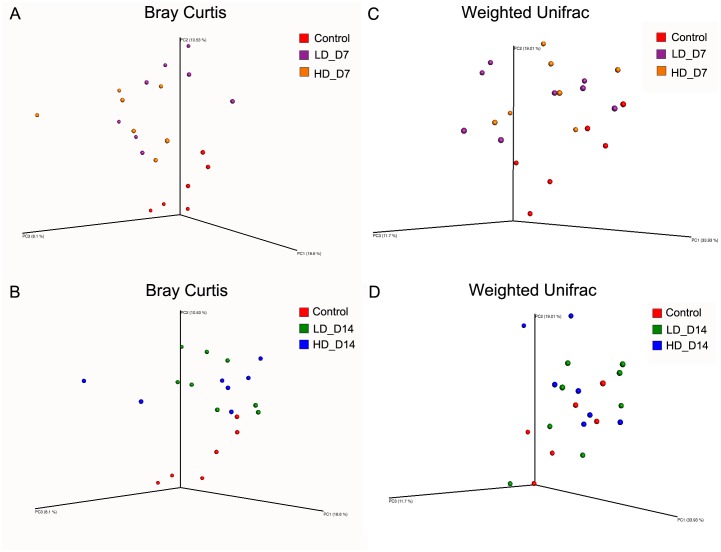
Three-dimensional principal coordinate analysis obtained with Bray Curtis distances (A and B) or weighted UniFrac distance matrix (C and D) of the salmon skin bacterial communities from day 7 infection (A and C) and day 14 infection (B and D). Each dot represents one individual fish. LD_D7: low-dose infected day 7; LD_D14: low-dose infected day 14; HD_D7: high-dose infected day 7; HD_D14: high-dose infected day 14.

These changes were further examined in beta diversity analyses using Bray Curtis distance (Fig 3A and 3B) and weighted Unifrac (Fig 3C and 3D). Principal coordinate analysis (PCoA) shows that, despite inter-individual variability within the control group (a common finding in teleost skin microbial communities [27, 28]), SAV3-infected samples clustered closer to each other, and that the distances between control and infected groups were greater at day 7 (Fig 3A) than at day 14 (Fig 3B).

### Both infection dose and time after infection determine the composition of the skin microbial community

ANOSIM statistical analyses revealed that infection treatment (control, low-dose, high-dose) (P value = 0.047) as well as time after infection (day 7 and day 14) (P value = 0.04) were significant factors determining the composition of the salmon skin microbial community.

### SAV-induced skin dysbiosis is characterized by loss of beneficial bacteria

**Fig 4:**
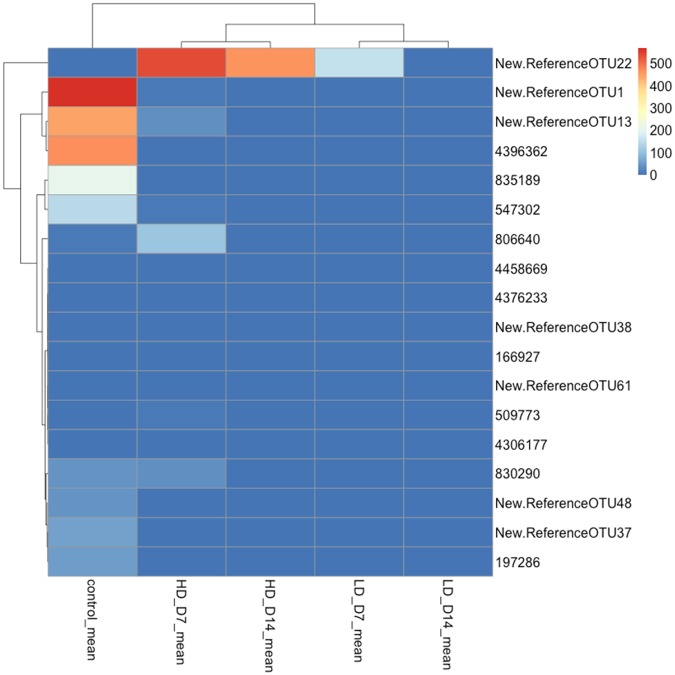
Heat map showing the mean relative abundances of OTUs that were found to be significantly different in control and SAV3 infected experimental groups. The taxonomic classification of each OTU number is shown in Table 2. LD_D7: low-dose infected day 7; LD_D14: low-dose infected day 14; HD_D7: high-dose infected day 7; HD_D14: high-dose infected day 14.

**Fig 5:**
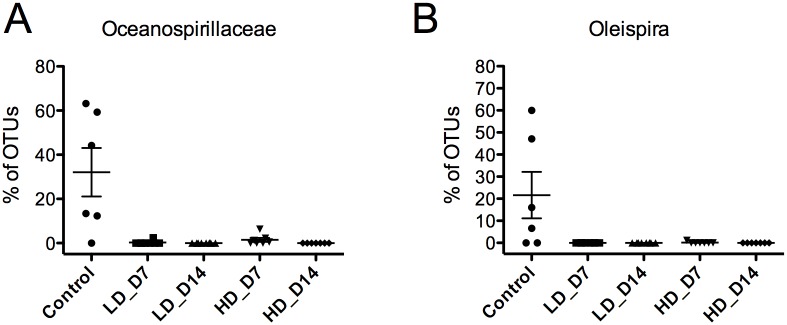
SAV3 infection results in losses of beneficial bacteria in Atlantic salmon skin. Percentage of total OTUs respresented by *Oceanospirillaceae* (A) and *Oleispira sp*. (B) in control and SAV-infected groups. The P values obtained in the statistical analysis are shown in Table 2. LD_D7: low-dose infected day 7; LD_D14: low-dose infected day 14; HD_D7: high-dose infected day 7; HD_D14: high-dose infected day 14.

We next determined whether SAV infection resulted in the absence of protective or physiologically important bacteria. A heat map of the 18 OTUs with significantly different abundances in each experimental group is shown in Fig 4. Out of these 18 OTUs, 14 belonged to the phylum Proteobacteria, 2 belonged to the phylum Firmicutes and 2 to the phylum Bacteroidetes. Among the OTUs that showed high abundance in controls, and low abundance in SAV-infected groups, we observed that the *Oceanospirillaceae* family, which represented ~ 32% of the bacterial community in controls, was practically absent in infected samples (Fig 5A and Table 2). Within this family, the genus *Oleispira* sp., known to be involved in the smoltification process of salmon [29], contributed to ~ 21% of the skin bacterial community in controls but was absent in all infected groups (Fig 5B and Table 2). Additionally, the abundances of *Enterobacteriaceae* and *Pseudoalteromonas sp*., both members of Gammaproteobacteria were present in controls, while were absent in the SAV-infected groups except for the high-dose day 7 group which had a similar abundance of *Pseudoalteromonas sp*. to the control group (Table 2 and Fig 4).

### SAV-induced skin dysbiosis is characterized by expansion of potentially pathogenic taxa

**Fig 6:**
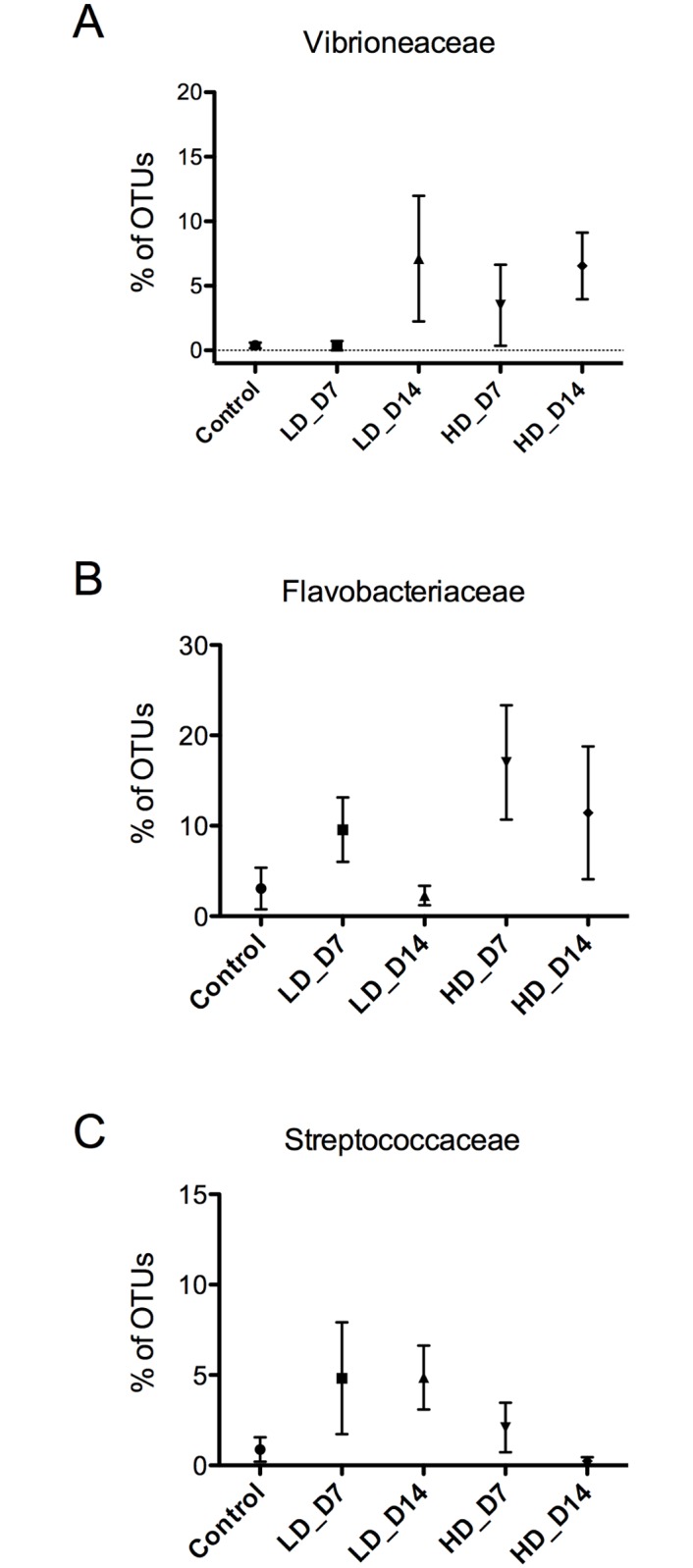
SAV3 infection results in increased abundance of opportunistic pathogens. Percentage of total OTUs respresented by *Vibrionaceae* (A), *Flavobacteriaceae* (B) and *Streptococcaceae* (C) in control and SAV-infected groups. The P values obtained in the statistical analysis are shown in Table 2. LD_D7: low-dose infected day 7; LD_D14: low-dose infected day 14; HD_D7: high-dose infected day 7; HD_D14: high-dose infected day 14.

**Fig 7:**
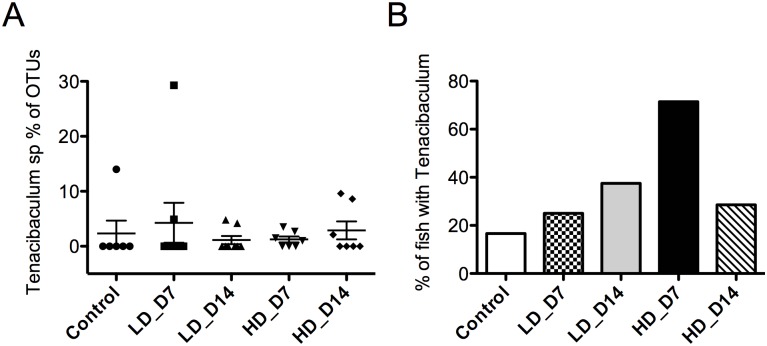
SAV3 infection results in increased abundance of *Tenacibaculum sp*. A) Percentage of total OTUs represented by *Tenacibaculum sp*. in control and SAV-infected groupsB) Percentage of fish containing *Tenacibaculum sp*. in their skin bacterial community in control and SAV-infected groups. LD_D7: low-dose infected day 7; LD_D14: low-dose infected day 14; HD_D7: high-dose infected day 7; HD_D14: high-dose infected day 14.

Among the 18 OTUs with abundances that were significantly different among experimental groups, we found significantly increased abundances of members of the families *Vibrionaceae*, *Flavobacteriaceae* and *Streptococcaceae sp*. (Table 2, Fig 6A–6C). Among *Flavobacteriaceae* members, *Tenacibaculum sp*. abundance increased from 2.5% of the skin microbial community in controls to 3.9% of in the high-dose day 14 group (Fig 7A). Additionally, examining the number of *Tenacibaculum sp*. positive individuals per experimental group, we found an increasing presence of this pathogen with viral infection dose and time. While only one of 6 (16.7%) individuals was positive for *Tenacibaculum sp*. in the control group, 2 out of 8 (25%) and 3 of 8 (37.5%) individuals were positive in the low-dose at day 7 and 14 respectively. Finally, 5 out of 7 (71.4%) and 2 of 7 (28.6%) individuals were positive for *Tenacibaculum sp*. in the high-dose group at day 7 and 14, respectively (Fig 7E). We did not observe any changes in the health status of animals that were *Tenacibaculum sp*. positive within the experimental period. A BLAST search of the *Tenacibaculum* OTU sequence revealed the highest level of similarity (100% identity) with the 16s rRNA gene sequence of *Tenacibaculum ovolyticum* strain da5A-8, a deep-sea fish pathogen with virulence factors similar to other *Tenacibaculum sp*. [30].

### Salmon skin dysbiosis is not correlated with SAV3 viral copy numbers in the heart

In order to know if there was a correlation between skin microbial community composition and viral load in infected individuals, we quantified SAV loads in the salmon heart by qPCR. We detected SAV in heart of one fish from the low-dose day 7 group, two fish in the high-dose day 7 group and 6 fish in the high-dose day 14 group (Table 3). The highest viral loads were present in the high-dose day 14 group with three fish with >1.7 x 10^5^ viral copy numbers (Table 3). We next examined whether all infected samples clustered together in the PCoA analysis, however, infected samples did not cluster together (S1 Fig). Further examination of the individual OTUs in specimens that tested positive for SAV in the heart did not reveal any trends or specific bacterial taxa. These results indicated that presence of SAV in the heart could not be correlated to skin dysbiosis in Atlantic salmon.

**Table 3:** Presence of SAV-3 in the heart tissue of Atlantic salmon used in the present study. SAV-3 copy numbers were estimated by qPCR using the nsP1 gene.

Treatment	Day	Raw Ct value	Copy number of nsP1
Control	8	Undetermined	0
Control	8	Undetermined	0
Control	8	Undetermined	0
Control	8	Undetermined	0
Control	8	Undetermined	0
Control	8	Undetermined	0
Control	8	Undetermined	0
Control	8	Undetermined	0
Low dose	7	Undetermined	0
Low dose	7	Undetermined	9
Low dose	7	Undetermined	0
Low dose	7	Undetermined	0
Low dose	7	Undetermined	0
Low dose	7	Undetermined	0
Low dose	7	Undetermined	0
Low dose	7	Undetermined	0
Low dose	14	Undetermined	0
Low dose	14	Undetermined	0
Low dose	14	Undetermined	0
Low dose	14	Undetermined	0
Low dose	14	33.8	78
Low dose	14	Undetermined	0
Low dose	14	Undetermined	0
Low dose	14	Undetermined	0
High dose	7	Undetermined	0
High dose	7	Undetermined	0
High dose	7	33.4	104
High dose	7	34.6	45
High dose	7	21.8	312000
High dose	7	28.6	2880
High dose	7	19.1	1990000
High dose	7	24.9	38000
High dose	14	Undetermined	0
High dose	14	30.2	985
High dose	14	34.8	40
High dose	14	Undetermined	0
High dose	14	35.4	26
High dose	14	25.8	19700
High dose	14	Undetermined	0
High dose	14	22.7	172000

## Discussion

Animal mucosal surfaces coexist with millions of microorganisms including bacteria, fungi and viruses. The harmonious relationship between host and microbiota can be disrupted by several environmental factors as well as host-intrinsic factors leading to dysbiosis. Pathogens are one of the factors that can tip the balance of host-microbiota relationships. Yet, how viral infections influence host microbial communities is still poorly understood.

The present study reveals important shifts in the skin bacterial community composition of Atlantic salmon in response to infection with SAV3. SAV poses serious economic losses to the salmon farming industry worldwide [17, 31, 32, 33, 34, 35]. Although the route of entry of SAV is currently unknown, previous studies have successfully infected salmon post-smolts with SAV by immersion challenge [21], mimicking natural infections in seawater salmon cages. As SAV is shed from infected individuals via the feces and skin mucus [20]. the skin of SAV-individuals was exposed to the virus not only on the day of the experimental infection, but potentially in a continuous fashion throughout the length of the experiment. SAV shedding has been shown to last for a period of 4 to 21 days [21]. Thus, shedding of infective viral particles into the water from individuals that were infected at the first exposure (onset of the experiment) raises the possibility of one initial exposure followed by several cycles of re-exposure to the virus. The latter may partially explain the lack of a direct correlation between SAV load in the heart and the composition of the skin microbiome. Nevertheless, our findings are in line with previous research showing that SAV viral loads in target organs such as the heart do not correlate with viral loads in non-target organs [24].

The states of dysbiosis identified in the low- and high-dose infected groups were not identical. Whereas the low-dose infected groups showed an overall loss of bacterial diversity, the high-dose infected groups had a more diverse (although not significant) skin microbiome. Very little is known about SAV viral loads at mucosal sites such as the skin or how the host mucosal immune system responds to SAV experimental infections. Our results suggest that different magnitudes in the mucosal immune response of the host to the virus may result in different skin microbial compositions, although this hypothesis needs to be tested. We speculate that the high-dose group mounted a stronger skin mucosal immune response that may have led to undesired skin colonization of taxa present in the water, including potential pathogens. Additionally, high-dose infected individuals may shed more SAV particles through the skin, which in turn, likely causes more tissue damage and altered local immune responses that in low-dose infected individuals. Our data further support the previously suspected complexity of the interactions between pathogens, host and the microbiota [36]. Future studies should address whether viral load in the skin correlates with the state of skin dysbiosis reported in this study and whether skin immune responses against the virus correlate with the observed changes in the skin microbiome of infected fish.

We found that both SAV3 infection dose and time of infection are strong predictors of the skin salmon microbiota. We did not perform microbiome analysis of tank water samples, and therefore we cannot rule out that the microbial composition of the seawater varied among treatment tanks and impacted the salmon skin microbiome. However, since the inlet water was mixed to the right temperature in one tank in the same room and then further distributed to all tanks, it is very unlikely that there were any external environmental factors or differences in the microbial composition of the water supplied to the treatment groups.

One the most notable changes in SAV infected skin microbial communities was the loss of Proteobacteria abundance. Proteobacteria are the predominant phylum in the skin microbiome of teleosts [28, 37, 38]. Importantly, several skin Proteobacteria isolates from salmonids have been shown to have inhibitory effects against bacterial and fungal pathogens [28, 38]. Moreover, Proteobacteria also dominate the human skin microbiome [39] and have been suggested to play a role in managing opportunistic bacteria and regulating host-environment relationships [40]. This information is in agreement with the observed expansions of *Flavobacteriaceae* and other known opportunistic taxa [41] in SAV-infected fish with lowered Proteobacteria abundance.

The loss of Proteobacteria abundance was primarily the result of a complete loss of *Oleispira sp*., a genus known to dominate the skin microbial community of salmon [29]. Importantly, *Oleispira* sp. abundance was found to increase dramatically in salmon skin as a result of smoltification [29]. Salmon are particularly susceptible to SAV at the time of transfer from freshwater to seawater. Stress induced by smoltification has long been linked to suppressed immune responses and increased disease susceptibility [42, 43]. We recorded absence of *Oleispira sp*. in all SAV-infected groups regardless of infection dose or time, indicating that this bacterial species was not able to recolonize the host during the duration of the experiment. Although the specific physiological contribution of *Oleispira sp*. to the salmon smoltification process is currently unknown, our findings suggest important inhibitory effects on SAV3 by *Oleispira sp*. present in the skin. Further studies should investigate if decreased *Oleispira sp*. abundances can contribute towards differences in disease susceptibility of salmon upon seawater transfer.

Previous studies have identified correlations between bacterial or parasitic infection and the microbiota in fish. Our results are in line with those found in a study in which *Aeromonas salmonicida* infection was associated with dominance shifting to opportunistic pathogens on the skin and mucus of Atlantic salmon [9]. On the other hand, metazoan parasite loads in the gut of tropical reef fish are negatively correlated with the presence of opportunistic bacteria in their gut microbiota, suggesting a protective role for metazoan parasites against occurrence of pathogenic bacteria in the fish gastrointestinal tract [7]. In the present study, we observed increased incidence and intensity of *Tenacibaculum sp*. in the skin of SAV3-infected post-smolts, particularly in the high-dose experimental groups. *Tenacibaculum sp*. has been described as a member of the salmon skin microbial community [29]. *Tenacibaculum sp*. is also the causative agent of winter ulcers in Atlantic salmon [44]. Although it has been identified as a primary pathogen of fish, this bacterium generally infects fish with mechanically abraded skin [45]. Viruses can augment the adhesion of secondary pathogenic bacteria to epithelial surfaces by increasing the expression of receptors for bacterial pathogens [46]. Thus, it is possible that SAV infection changes the expression of receptors for *Tenacibaculum sp*. in salmon skin epithelial cells. This finding suggests a possible link between SAV infection and *Tenacibaculum sp*. skin infections in salmon. Since our observations show a higher presence of *Tenacibaculum* in SAV infected fish in a dose-dependent fashion, this link deserves to be further investigated for a better understanding of the dynamics of disease outbreaks in salmon farms.

## Conclusions

SAV3 infection results in skin dysbiosis in Atlantic salmon characterized most prominently by the loss of Proteobacteria. The loss of microbial balance caused by viral infections such as SAV3 likely impacts the health of the fish host, rendering the fish more susceptible to secondary infections by opportunistic bacterial pathogens present in the environment or within the host indigenous microbial reservoir.

## Supporting information

S1 Fig Three-dimensional principal coordinate analysis obtained with weighted Unifrac distances of Atlantic salmon microbiome samples that were positive for SAV3 in the heart tissue by qPCR. LD_D7: low-dose infected day 7; HD_D7: high-dose infected day 7; HD_D14: high-dose infected day 14.
(PDF)

## References

[1] Schmidt V, Amaral-Zettler L, Davidson J, Summerfelt S, Good C. Influence of fishmeal-free diets on microbial communities in Atlantic Salmon (*Salmo salar*) recirculation aquaculture systems. Appl Environ Microbiol. 2016; 82(15): 4470–4481. 10.1128/AEM.00902-16 27129964 PMC4984271

[2] Gaulke CA, Barton CL, Proffitt S, Tanguay RL, Sharpton TJ. Triclosan exposure is associated with rapid restructuring of the microbiome in adult zebrafish. PLoS One. 2016; 11(5): e0154632 10.1371/journal.pone.0154632 27191725 PMC4871530

[3] Eichmiller JJ, Hamilton MJ, Staley C, Sadowsky MJ, Sorenson PW. Environment shapes the fecal microbiome of invasive carp species. Microbiome. 2016; 4: 44 10.1186/s40168-016-0190-1 27514729 PMC4981970

[4] Sylvain F, Cheaib B, Llewellyn M, Correia TG, Fagundes DB, Val AL, et al pH drop impacts differentially skin and gut microbiota of the Amazonian fish tambaqui (*Colossoma macropomum*). Sci Rep. 2016; 6: 32032 10.1038/srep32032 27535789 PMC4989189

[5] Llewellyn MS, Boutin S, Hoseinifar SH, Derome N. Teleost microbiomes: the state of the art in their characterization, manipulation and importance in aquaculture and fisheries. Front Microbiol. 2014; 5: 207 10.3389/fmicb.2014.00207 24917852 PMC4040438

[6] Honda K, Littman DR. The microbiome in infectious disease and inflammation. Annu Rev Immunol. 2012; 30: 759–795. 10.1146/annurev-immunol-020711-074937 22224764 PMC4426968

[7] Hennersdorf P, Kleinertz S, Theisen S, Abdul-Aziz MA, Mrotzek G, Palm HW, et al Microbial diversity and parasitic load in tropical fish of different environmental conditions. PLoS ONE. 2016; 11(3): e0151594 10.1371/journal.pone.0151594 27018789 PMC4809571

[8] Toranzo AE, Novoa B, Romalde JL, Nunez S, Devesa S, Marino E, et al Microflora associated with healthy and diseased turbot (*Scophthalmus maximus*) from three farms in northwest Spain. Aquaculture. 1993; 114: 189–202.

[9] Cipriano RC, Dove A. Far from superficial: microbial diversity associated with the dermal mucus of fish In: Cipriano RC, Schelkunov I, editors. Health and Diseases of Aquatic Organisms: Bilateral Perspectives. East Lansing: MSU Press; 2011 pp. 156–167.

[10] Robinson CM, Pfeiffer JK. Viruses and the Microbiota. Annu Rev Virol. 2014; 1: 55–69. 10.1146/annurev-virology-031413-085550 25821837 PMC4373533

[11] David R. Viral infection: The gut microbiota: friend or foe? Nat Rev Microbiol. 2011; 9: 831 10.1038/nrmicro2691 22048739

[12] Wilks J, Golovkina T. Influence of microbiota on viral infections. PLoS Pathog. 2012; 8(5): e1002681 10.1371/journal.ppat.1002681 22615558 PMC3355081

[13] Kuss SK, Best GT, Etheredge CA, Pruijssers AJ, Frierson JM, et al Intestinal microbiota promote enteric virus replication and systemic pathogenesis. Science. 2011; 334 (6053): 249–252. 10.1126/science.1211057 21998395 PMC3222156

[14] Kane M, Case LK, Kopaskie K, Koslova A, MacDearmid C, Chervonsky AV, et al Successful transmission of a retrovirus depends on the commensal microbiota. Science. 2011; 334 (6053): 245–249. 10.1126/science.1210718 21998394 PMC3519937

[15] Vujkovic-Cvijin I, Dunham RM, Iwai S, Maher MC, Albright RG, Broadhurst MJ, et al Dysbiosis of the gut microbiota is associated with HIV disease progression and tryptophan catabolism. Sci Transl Med. 2013; 5(193): 193–191. 10.1126/scitranslmed.3006438 PMC4094294 23843452

[16] Deriu E, Boxx GM, He X, Pan C, Benavidez SD, Cen L, et al Influenza virus affects intestinal microbiota and secondary salmonella infection in the gut through type I interferons. PLoS Pathog. 2016; 12(5): e1005572 10.1371/journal.ppat.1005572 27149619 PMC4858270

[17] McLoughlin MF, Graham DA. Alphavirus infections in salmonids—a review. J Fish Dis. 2007; 30 (9): 511–531. 10.1111/j.1365-2761.2007.00848.x 17718707

[18] Jansen MD, Taksdal T, Wasmuth MA, Gjerset B, Brun E, Olsen AB, et al Salmonid alphavirus (SAV) and pancreas disease (PD) in Atlantic salmon, *Salmo salar L*., in freshwater and seawater sites in Norway from 2006 to 2008. J Fish Dis. 2010; 33 (5): 391–402. 10.1111/j.1365-2761.2009.01131.x 20158578

[19] Graham DA, Frost P, McLaughlin K, Rowley HM, Gabestad I, Gordon A, et al A comparative study of marine salmonid alphavirus subtypes 1–6 using an experimental cohabitation challenge model. J Fish Dis. 2011; 34 (4), 273–286. 10.1111/j.1365-2761.2010.01234.x 21294751

[20] Graham DA, Brown A, Savage P, Frost P. Detection of salmon pancreas disease virus in the faeces and mucus of Atlantic salmon, *Salmo salar* L., by real-time RT-PCR and cell culture following experimental challenge. J Fish Dis. 2012; 35 (12): 949–951. 10.1111/j.1365-2761.2012.01427.x 22924477

[21] Jarungsriapisit J, Moore LJ, Taranger GL, Nilsen TO, Morton HC, Fiksdal IU, et al Atlantic salmon (*Salmo salar* L.) post-smolts challenged two or nine weeks after seawater-transfer show differences in their susceptibility to salmonid alphavirus subtype 3 (SAV3). Virol J. 2016; 13:66 10.1186/s12985-016-0520-8 27068518 PMC4827186

[22] Jarungsriapisit J, Moore LJ, Mæhle S, Skår C, Einen AC, Fiksdal IU, et al Relationship beteween viral dose and outcome of infection in Atlantic salmon, *Salmo salar L*., post-smolts bath-challenged with salmonid alphavirus subtype 3. Vet Res. 2016; 47(1):102 10.1186/s13567-016-0385-2 27760562 PMC5069985

[23] Mitchell KR, Takacs-Vesbach CD. A comparison of methods for total community DNA preservation and extraction from various thermal environments. J Ind Microbiol Biotechnol. 2008; 35:1139–1147. 10.1007/s10295-008-0393-y 18633656

[24] Andersen L, Hodneland K, Nylund A. No influence of oxygen levels on pathogenesis and virus shedding in Salmonid alphavirus (SAV)-challenged Atlantic salmon (*Salmo salar L*.). Virol J. 2010; 7 (198): 1–14. 20727205 10.1186/1743-422X-7-198 PMC2936311

[25] Caporaso JG, Kuczynski J, Stombaugh J, Bittinger K, Bushman FD, Costello EK, et al QIIME allows analysis of high-throughput community sequencing data. Nat Methods. 2010; 7: 335–336. 10.1038/nmeth.f.303 20383131 PMC3156573

[26] Giardine B, Riemer C, Hardison RC, Burhans R, Elnitski L, Shah P, et al Galaxy: A platform for interactive large-scale genome analysis. Genome Res. 2005; 15(10): 1451–1455. 10.1101/gr.4086505 16169926 PMC1240089

[27] Boutin S, Sauvage C, Bernatchez L, Audet C, Derome N. Inter-individual variations of the fish skin microbiota: host genetics basis of mutualism? PLoS ONE. 2014; 9(7): e102649 10.1371/journal.pone.0102649 25068850 PMC4113282

[28] Lowrey L, Woodhams DC, Tacchi L, Salinas I. Topographical mapping of the rainbow trout (*Oncorhynchus mykiss*) microbiome reveals a diverse bacterial community with antifungal properties in the skin. Appl Environ Microbiol. 2015; 81 (19): 6915–6925. 10.1128/AEM.01826-15 26209676 PMC4561705

[29] Lokesh J, Kiron V. Transition from freshwater to seawater reshapes the skin-associated microbiota of Atlantic salmon. Sci Rep. 2016; 6 (19707). 10.1038/srep19707 PMC4726331 26806545

[30] Teramoto M, Zhai Z, Komatsu A, Shibayama K, Suzuki M. Genome sequence of the psychrophilic bacterium *Tenacibaculum ovolyticum* strain da5A-8 isolated from deep seawater. Genome Announc. 2016; 4(3): e00644–16. 10.1128/genomeA.00644-16 27365358 PMC4929521

[31] Borzym E, Maj-Paluch J, Stachnik M, Matras M, Reichert M. First laboratory confirmation of salmonid alphavirus type 2 (SAV2) infection in Poland. Bull Vet Inst Pulawy. 2014; 58 (3): 341–345.

[32] Bergmann SM, Fichtner D, Riebe R, Castric J. First isolation and identification of Sleeping Disease Virus (SDV) in Germany. Bull Eur Ass Fish Pathol. 2008; 28 (4): 148–156.

[33] Graham DA, Rowley HM, Fringuelli E, Bovo G, Manfrin A, McLoughlin MF, et al First laboratory confirmation of salmonid alphavirus infection in Italy and Spain. J Fish Dis. 2007; 30 (9): 569–572. 10.1111/j.1365-2761.2007.00826.x 17718711

[34] Schmidt-Posthaus H, Diserens N, Hjortaas MJ, Knuesel R, Hirschi R, Taksdal T. First outbreak of sleeping disease in Switzerland: disease signs and virus characterization. Dis Aquat Organ. 2014; 111 (2): 165–171. 10.3354/dao02766 25266904

[35] Smrzlic IV, Kapetanovic D, Valic D, Teskeredzic E, McLoughlin MF, Fringuelli E. First laboratory confirmation of sleeping disease virus (SDV) in Croatia. Bull Eur Ass Fish Path. 2013; 33 (3): 78–83.

[36] Boutin S, Bernatchez L, Audet C, Derome N. Network analysis highlights complex interactions between pathogen, host and commensal microbiota. PLoS ONE. 2013; 8:e84772 10.1371/journal.pone.0084772 24376845 PMC3871659

[37] Larsen A, Tao Z, Bullard SA, Arias CR. Diversity of skin microbiota of fishes: evidence for host species specificity. FEMS Microbiol Ecol. 2013; 85(3): 483–494. 10.1111/1574-6941.12136 23607777

[38] Boutin S, Bernatchez L, Audet C, Derôme N. Antagonistic effect of indigenous skin bacteria of brook charr (*Salvelinus fontinalis*) against *Flavobacterium columnare* and *F*. *psychrophilum*. Vet Microbiol. 2012; 155(2–4): 355–61. 10.1016/j.vetmic.2011.09.002 21958747

[39] Cosseau C, Romano-Bertrand S, Duplan H, Lucas O, Ingrassia I, Pigasse C, et al *Proteobacteria* from the human skin microbiota: Species-level diversity and hypotheses. One Health. 2016; 2: 33–41. 28616476 10.1016/j.onehlt.2016.02.002 PMC5441325

[40] Grice EA, Segre JA. The skin microbiome. Nat Rev Microbiol. 2011; 9(4): 244–253. 10.1038/nrmicro2537 21407241 PMC3535073

[41] Derome N, Gauthier J, Boutin S, Llewellyn M. Bacterial opportunistic pathogens of fish In: Hurst CJ, editor. The Rasputin effect, when commensals and symbionts become parasitic. Switerland: Springer International Publishing; 2016 pp. 81–108.

[42] Eggset G, Mortensen A, Johansen L, Sommer A. Susceptibility to furunculosis, cold water vibriosis, and infectious pancreatic necrosis (IPN) in post-smolt Atlantic salmon (*Salmo salar* L.) as a function of smolt status by seawater transfer. Aquaculture. 1997; 158 (3–4): 179–191.

[43] Mesa MG, Maule AG, Poe TP, Schreck CB. Influence of bacterial kidney disease on smoltification in salmonids: is it a case of double jeopardy? Aquaculture. 1999; 174: 25–41.

[44] Olsen AB, Nilsen H, Sandlund N, Mikkelsen H, Sørum H, Colquhoun DJ. *Tenacibaculum sp*. associated with winter ulcers in sea-reared Atlantic salmon *Salmo salar*. Dis Aquat Org. 2011; 94: 189–199. 10.3354/dao02324 21790066

[45] Rahman T, Suga K, Kanai K, Sugihara Y. Infection kinetics of Tenacibaculum maritimum on abraded skin of Japanese flounder *Paralichthys olivaceus*. Fish Pathol. 2015; 50 (2): 44–52.

[46] Avadhanula V, Rodriguez CA, DeVincenzo JP, Wang Y, Webby RJ, Glen C. Ulett GC, et al Respiratory viruses augment the adhesion of bacterial pathogens to respiratory epithelium in a viral species-and cell type- dependent manner. J Virol. 2006; 80 (4): 1629–1636. 10.1128/JVI.80.4.1629-1636.2006 16439519 PMC1367158

