# Supplementary material for: Salmonid alphavirus infection causes skin dysbiosis in Atlantic salmon (Salmo salar L.) post-smolts
Source: PLoS One. 2017 Mar 6;12(3):e0172856. doi: 10.1371/journal.pone.0172856 (PMC5338768; doi:10.1371/journal.pone.0172856)

S1 Fig: Three-dimensional principal coordinate analysis obtained with weighted Unifrac distances of Atlantic salmon microbiome samples that were positive for SAV3 in the heart tissue by qPCR. LD_D7: low-dose infected day 7; HD_D7: high-dose infected day 7; HD_D14: high-dose infected day 14.

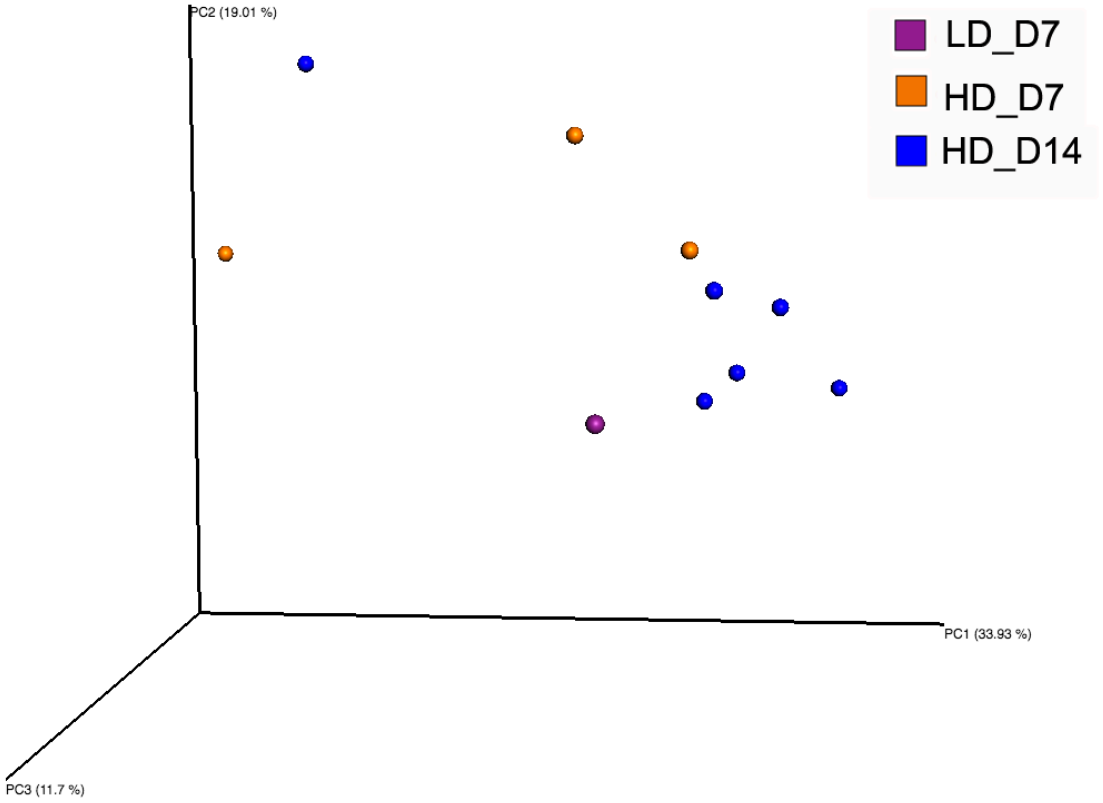

Supplement: S1 Fig — LD_D7: low-dose infected day 7; HD_D7: high-dose infected day 7; HD_D14: high-dose infected day 14. (PDF) [file pone.0172856.s001.pdf]
